# Enhanced Muscle Strength in Dyslipidemic Mice and Its Relation to Increased Capacity for Fatty Acid Oxidation

**DOI:** 10.3390/ijms222212251

**Published:** 2021-11-12

**Authors:** Marta Tomczyk, Alicja Braczko, Patrycja Jablonska, Adriana Mika, Kamil Przyborowski, Agata Jedrzejewska, Oliwia Krol, Filip Kus, Tomasz Sledzinski, Stefan Chlopicki, Ewa M. Slominska, Ryszard T. Smolenski

**Affiliations:** 1 Department of Biochemistry, Medical University of Gdansk, 80-211 Gdansk, Poland; alicja.braczko@gumed.edu.pl (A.B.); patrycja.jablonska@gumed.edu.pl (P.J.); agata.jedrzejewska@gumed.edu.pl (A.J.); oliwia.krol@gumed.edu.pl (O.K.); kusfi@gumed.edu.pl (F.K.); eslom@gumed.edu.pl (E.M.S.); 2 Department of Pharmaceutical Biochemistry, Medical University of Gdansk, 80-211 Gdansk, Poland; adriana.mika@gumed.edu.pl (A.M.); tomasz.sledzinski@gumed.edu.pl (T.S.); 3 Jagiellonian Centre for Experimental Therapeutics, 30-348 Krakow, Poland; kamil.przyborowski@jcet.eu (K.P.); stefan.chlopcki@jcet.eu (S.C.); 4 Intercollegiate Faculty of Biotechnology UG-MUG, Medical University of Gdansk, 80-210 Gdansk, Poland; 5 Chair of Pharmacology, Faculty of Medicine, Jagiellonian University Medical College, 30-688 Krakow, Poland

**Keywords:** skeletal muscle, metabolic disorders, dyslipidemia, mitochondria

## Abstract

Dyslipidemia is commonly linked to skeletal muscle dysfunction, accumulation of intramyocellular lipids, and insulin resistance. However, our previous research indicated that dyslipidemia in apolipoprotein E and low-density lipoprotein receptor double knock-out mice (ApoE/LDLR -/-) leads to improvement of exercise capacity. This study aimed to investigate in detail skeletal muscle function and metabolism in these dyslipidemic mice. We found that ApoE/LDLR -/- mice showed an increased grip strength as well as increased troponins, and Mhc2 levels in skeletal muscle. It was accompanied by the increased skeletal muscle mitochondria numbers (judged by increased citrate synthase activity) and elevated total adenine nucleotides pool. We noted increased triglycerides contents in skeletal muscles and increased serum free fatty acids (FFA) levels in ApoE/LDLR -/- mice. Importantly, Ranolazine mediated inhibition of FFA oxidation in ApoE/LDLR -/- mice led to the reduction of exercise capacity and total adenine nucleotides pool. Thus, this study demonstrated that increased capacity for fatty acid oxidation, an adaptive response to dyslipidemia leads to improved cellular energetics that translates to increased skeletal muscle strength and contributes to increased exercise capacity in ApoE/LDLR -/- mice.

## 1. Introduction

Skeletal muscle is a heterogenic, dynamic, and flexible structure that is based on the arrangement of muscle fibers and associated connective tissue [1]. Recent studies underlined the role of the extracellular matrix in maintaining the integrity of skeletal muscle, biochemical signaling, or playing a vital role during myogenesis [2,3]. Muscle fibers are characterized by significant variability in the biochemical, mechanical, and metabolic phenotypes. The presence of fibers with different properties in the same muscle may reflect an adaptation to distinctive patterns of physical activity or genetic and metabolic diversity [1]. The most frequently used classification revealed the existence of four fiber types in mammalian skeletal muscles identified by the presence of specific myosin heavy chain isoforms: mitochondria-rich slow type 1 fiber, mitochondria-rich fast 2A, and 2X fibers, and mitochondria-poor fast 2B fibers [4,5]. Interestingly, different types of muscle were characterized also by the specific distribution of the two mitochondrial isocitrate dehydrogenases, IDH2 and IDH3 [6].

Regardless of the skeletal muscle type, they require energy in the form of adenosine triphosphate (ATP). There are three basic energy pathways in which skeletal muscle fiber may gain ATP: muscles stores of ATP and phosphocreatine (PCr), anaerobic glycolysis, and oxidative phosphorylation. It is well known that carbohydrates (plasma glucose and muscle glycogen stores) and fatty acids (plasma free fatty acids and muscle triglycerides stores) are the two main metabolic fuels utilized by the muscle cell to produce ATP [7]. In high intensities, muscle actions are mainly fueled by muscle glycogen stores. Diversely, low intensity and long duration exercise utilize the metabolism of free fatty acids for most of the energy needs. Nevertheless, the use of appropriate substrates may also depend on their availability and metabolic substrate preference.

Recently, we highlighted that the mouse model of dyslipidemia- apolipoprotein E and low-density lipoprotein receptor double knock-out mice (ApoE/LDLR -/-), exhibit metabolic preference switch from glucose to free fatty acids, not only in cardiac but also in skeletal muscle. Interestingly, we indicated that changes were accompanied by increased exercise capacity despite the presence of endothelial dysfunction and atherosclerosis [8,9,10,11]. We suggested that robust compensatory mechanisms in coronary circulation involving PGI2- and NO-pathways might have been responsible for surprising exercise capacity counterbalancing coronary atherosclerosis [8,11] that was also associated with compensatory up-regulation of PGI2-dependent anti-platelet mechanisms in ApoE/LDLR -/- mice. However, in previous studies, we did not study skeletal muscle adaptation in detail focusing rather on cardiac metabolism [9]. Thus, in this study, we aimed to investigate in detail skeletal muscle function and metabolism in ApoE/LDLR -/- mice, as well as compare these changes with another mouse model of dyslipidemia, low-density lipoprotein receptor knock-out (LDLR -/-) mouse model. Moreover, we characterized the glycogen and triglycerides stores as well as levels of proteins involved in oxidative metabolism in mice’s skeletal muscle. Furthermore, to underpin the involvement of free fatty acids metabolism in those changes, we inhibited the free fatty acid oxidation within Ranolazine and analyzed animal exercise capacity. Additionally, we profiled the serum fatty acid content in ApoE/LDLR -/- double knock-out mice.

## 2. Results

### 2.1. Improved Grip Strength and Skeletal Muscle Troponins Content in ApoE/LDLR -/- Mice

**Figure 1:**
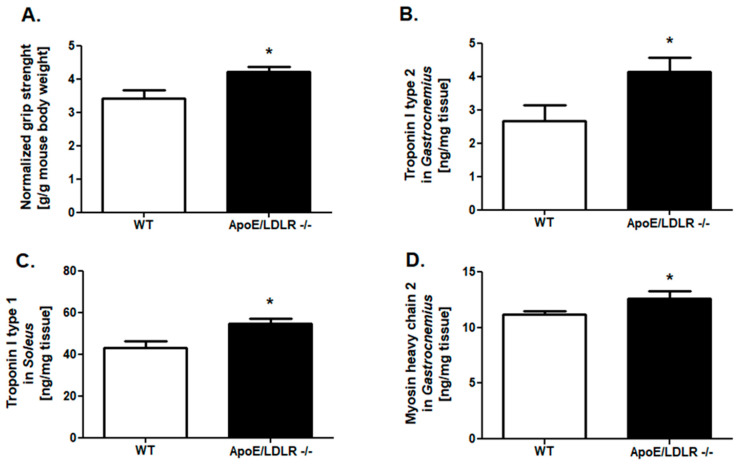
Improved forelimb grip strength, increased skeletal muscle troponins, and skeletal muscle myosin heavy chain 2 levels in ApoE/LDLR -/- mice. Normalized forelimb grip strength (maximum forelimb grip strength/g of body weight) (**A**), troponin I type 2 levels in *gastrocnemius* (fast skeletal muscle) (**B**), troponin I type 1 level in *soleus* (slow skeletal muscle) (**C**) and (**D**) myosin heavy chain 2 levels in *gastrocnemius* of control (WT), and ApoE/LDLR -/- mice. Results presented as mean ± SEM, *n*= 6–8, * *p* < 0.05.

**Figure 2:**
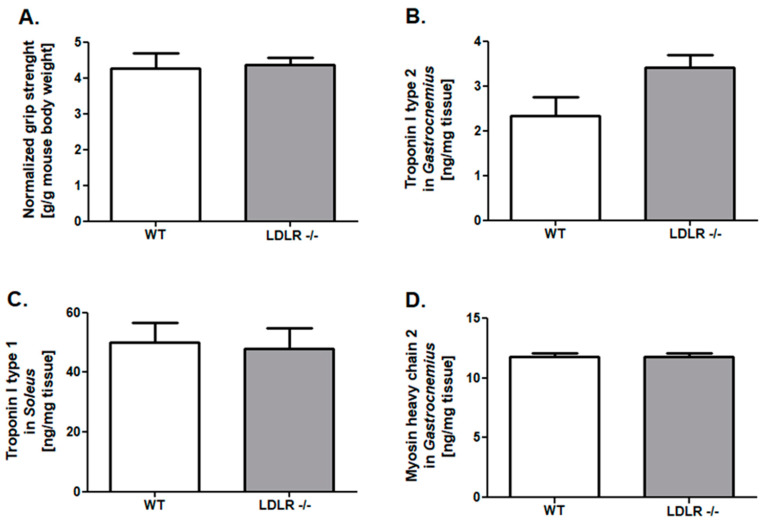
No changes in forelimb grip strength, skeletal muscle troponins, and myosin heavy chain 2 levels in LDLR -/- mice. Normalized forelimb grip strength (maximum forelimb grip strength/g of body weight) (**A**), troponin I type 2 levels in *gastrocnemius* (fast skeletal muscle) (**B**), troponin I type 1 level in *soleus* (slow skeletal muscle) (**C**) and myosin heavy chain 2 levels in *gastrocnemius* (**D**) of control (WT), LDLR -/- mice. Results presented as mean ± SEM, *n* = 6–8.

To investigate the skeletal muscle function of mouse models of dyslipidemia, we measured normalized grip strength in ApoE/LDLR -/-, LDLR -/-, and their WT littermates. It indicated higher values of this parameter in ApoE/LDLR -/- mice in comparison to control mice (Figure 1A). No changes were noted between LDLR -/- and control mice (Figure 2A).

The next step of our research was the examination of proteins levels characteristic for fast (*gastrocnemius*): troponin I type 2 and myosin heavy chain 2, as well as for slow (*soleus*) skeletal muscle such as troponin I type I. We noted elevated troponin I type 2 and myosin heavy chain 2 levels in *gastrocnemius* of ApoE/LDLR -/- relative to WT littermates (Figure 1C,E). Interestingly, we also observed an increased troponin I type 1 level in *soleus* isolated from ApoE/LDLR -/- mice (Figure 1D). Nevertheless, there were no significant changes in troponins (Type I 1 and 2) and myosin heavy chain 2 degrees in skeletal muscles of LDLR -/- relative to control mice (Figure 2C–E).

### 2.2. Compensatory Changes in Mitochondria in Skeletal Muscle in ApoE/LDLR -/- Mice 

**Figure 3:**
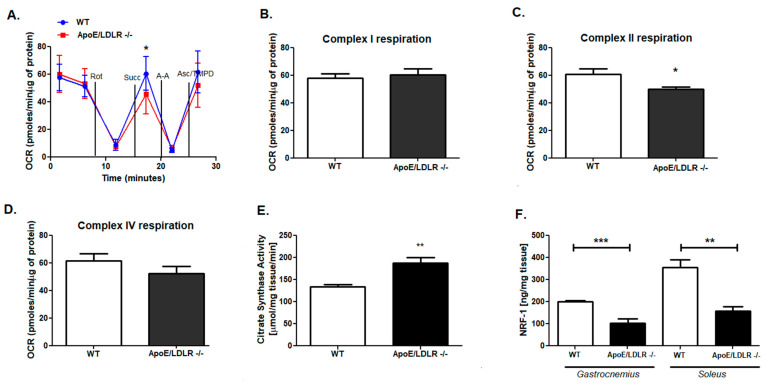
Increased citrate synthase activity despite accompanied derangements in mitochondrial complex II activity and nuclear respiratory factor 1 level in ApoE/LDLR -/- mice skeletal muscle. OCR data (**A**), complex I (**B**), II (**C**) and IV respiration in *soleus* (**D**). Mitochondrial citrate synthase activity in *soleus* (**E**) and nuclear respiratory factor 1 level in *gastrocnemius* and *soleus* (**F**) of WT and ApoE/LDLR -/- mice. Data presented as mean ± SEM, *n* = 3–7, * *p* < 0.05, ** *p* < 0.01, *** *p* < 0.001.

**Figure 4:**
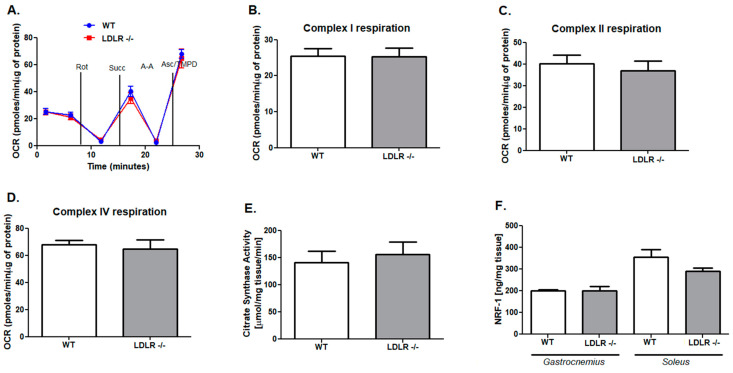
No changes in mitochondrial complexes respiration rates, citrate synthase activity, and nuclear respiratory factor 1 level in LDLR -/- mice skeletal muscle. OCR data (**A**), complex I (**B**), II (**C**) and IV (**D**) respiration in *soleus*. Mitochondrial citrate synthase activity in *soleus (***E**) and nuclear respiratory factor 1 levels in *gastrocnemius* and *soleus* (**F**) of LDLR -/- and WT mice. Data presented as mean ± SEM, *n* = 3–7.

The effectiveness of long and intensive exercise capacity depends on proper mitochondrial function and metabolism. Thus, we aimed to evaluate the mitochondrial oxidative chain complexes respiration in mitochondria-rich, red soleus muscles isolated from dyslipidemic mice as well as their WT littermates. There were no changes in complex I and complex IV respiration, while reduced complex II respiration and OCR value after succinate addition in ApoE/LDLR -/- relative to control were highlighted (Figure 3A–D). Moreover, we revealed that mitochondria isolated from soleus of LDLR -/- and WT mice had similar mitochondrial oxidative capacities. No changes were noted in OCR ratio and mitochondrial complexes activities (Figure 4A–D). Despite the skeletal muscle mitochondrial functionality, the overall skeletal muscle oxidative capacity depends also on mitochondria number. Thus, we measured the activity of citric synthase, the indicator of mitochondria amount in *soleus* of ApoE/LDLR -/-, LDLR -/-, and control mice. Interestingly, we noted elevated activity of this enzyme in ApoE/LDLR -/- relative to WT (Figure 3E). *Soleus* muscle isolated from LDLR -/- mice revealed no statistically significant differences in citric synthase activity in comparison to control mice (Figure 4E). 

Next, to address the reason for mitochondrial oxidative capacity derangements in ApoE/LDLR -/- skeletal muscle, we assessed the levels of one of the transcriptional factors involved in cellular respiration: nuclear respiratory factor 1 (NRF1). We found that despite no changes in levels of this compound in LDLR -/- mice skeletal muscle (*gastrocnemius* and *soleus*), another dyslipidemic mice model, ApoE/LDLR -/- exhibited intensive suppression on NRF1 levels in both white (*gastrocnemius*) and red (*soleus*) types of skeletal muscles (Figure 3F and Figure 4F).

### 2.3. Enhanced Adenine Nucleotides Pool Is Accompanied by an Elevation of Triglycerides and Depletion of Glycogen Stores in ApoE/LDLR -/- Mice Skeletal Muscle

**Figure 5:**
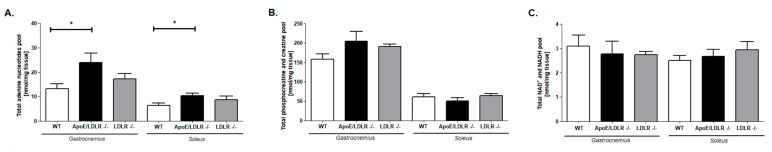
Elevated total adenine nucleotides pool in skeletal muscle of ApoE/LDLR -/- mice. Total adenine nucleotides (**A**), phosphocreatine and creatine (**B**), and NAD+ and NADH (**C**) pools in *gastrocnemius* and *soleus* of control (WT), ApoE/LDLR -/-, and LDLR -/-. Results presented as mean ± SEM, *n* = 4–8, * *p* < 0.05.

The further analysis tested whatever changes in mitochondria function and number in ApoE/LDLR -/- mice may affect overall skeletal muscle energy metabolism. Measurements of total creatine and phosphocreatine, total adenine nucleotides, and total nicotinamide dinucleotides (NAD+ and NADH) pools in *gastrocnemius* and *soleus* muscle were assessed. We noted that total adenine nucleotides pools were elevated in both of investigated skeletal muscle-*gastrocnemius* and *soleus* of ApoE/LDLR -/- mice (Figure 5A). On the other hand, there were no changes in total phosphocreatine and creatine pool and total NAD+ and NADH pools in ApoE/LDLR -/- skeletal muscles relative to WT mice (Figure 5B,C). As in previous experiments, we measured the same parameters also in LDLR -/- mice. Predictably, there were no changes in energy metabolism status parameters (total creatine and phosphocreatine, total adenine nucleotides, and total nicotinamide dinucleotides pools) in LDLR -/- skeletal muscle relative to WT (Figure 5A–C).

**Figure 6:**
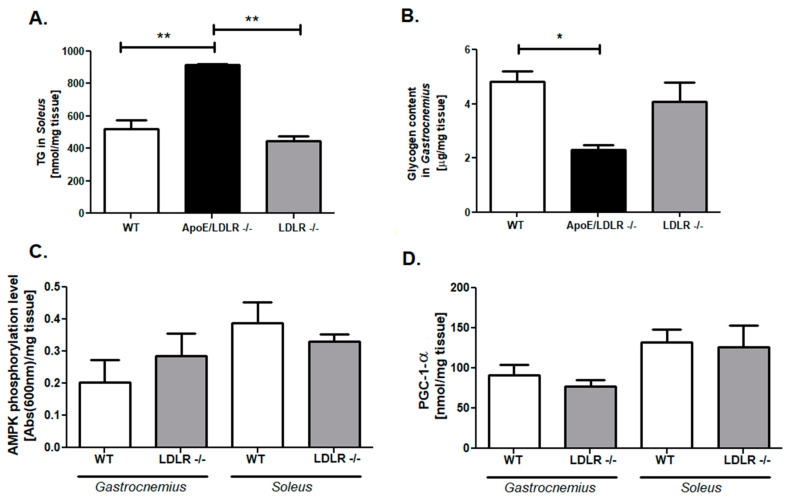
Elevated triglycerides stores in *soleus* and diminished glycogen content in *gastrocnemius* of ApoE/LDLR -/- but not LDLR -/- mice. Triglycerides (**A**) or glycogen (**B**) level in *gastrocnemius* from WT, ApoE/LDLR -/-, and LDLR -/- mice. AMP-activated protein kinase phosphorylation (**C**) and PGC-1α levels (**D**) in *gastrocnemius* and *soleus* of WT and LDLR -/- mice. Results presented as mean ± SEM, *n* = 5–8, * *p* < 0.05, ** *p* < 0.001.

To further investigate the metabolism we examined skeletal muscles triglycerides (TG) as well as glycogen stores. The main TG storages are red skeletal muscles, thus we examined TG levels in the *soleus* muscle. We found that ApoE/LDLR -/- mice *soleus* was characterized by higher TG content relative to WT littermates (Figure 6A). Interestingly, this was so far the only parameter that statistically distinguished ApoE/LDLR -/- and LDLR -/- mice. On the other hand, an investigation of glycogen stores in *gastrocnemius* revealed diminished glycogen content in ApoE/LDLR -/- mice in comparison to control (Figure 6B). There were no changes in those parameters between LDLR -/- and WT mice (Figure 6A,B). Nevertheless, to create and compare the overall metabolic characterization, we evaluated also levels of AMPK phosphorylation and PGC-1α in *gastrocnemius and soleus* from LDLR -/- mice, as we have done for ApoE/LDLR -/- mice in our previous work [9]. We noted no statistical changes in both of these parameters between LDLR -/- and WT mice skeletal muscle (Figure 6C,D).

### 2.4. Free Fatty Acids Elevation as a Key Factor for Skeletal Muscle Function Improvement in ApoE/LDLR -/- Mice Model

**Figure 7:**
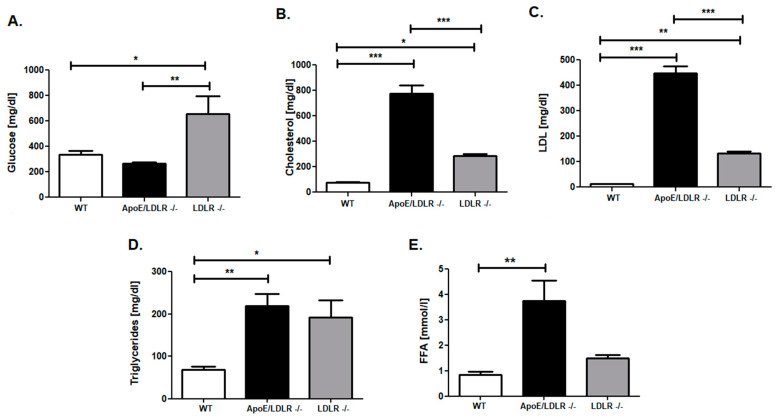
Profound alterations in blood lipid (ApoE/LDLR -/-) and glucose (LDLR -/-). Serum concentrations of glucose (**A**), cholesterol (**B**), LDL (**C**), triglycerides (**D**), and free fatty acid (**E**). Results presented as mean ± SEM, *n* = 6–8, * *p* < 0.05, ** *p* < 0.01, *** *p* < 0.001.

To unravel the origin of elevated triglyceride skeletal muscle stores in ApoE/LDLR -/-, we measured the lipid profile (cholesterol, LDL, triglycerides) and glucose levels in mouse models of dyslipidemia relative to control littermates (Figure 7). We found that ApoE/LDLR -/- mice were characterized by extensively elevated cholesterol and LDL levels in comparison to WT as well as LDLR -/- mice (Figure 7B,C). In the case of triglycerides concentration, we found that ApoE/LDLR -/- do not exhibit statistically significant changes in this parameter relative to LDLR -/-. Nevertheless, levels observed in both dyslipidemic mouse models were statistically higher in comparison to WT littermates (Figure 7D). Interestingly, glucose levels were increased in LDLR -/- when compared to control as well as ApoE/LDLR -/- mice (Figure 7A). Furthermore, ApoE/LDLR -/- mice exhibited massive elevation of serum-free fatty acid in comparison to WT (Figure 7E). 

**Figure 8:**
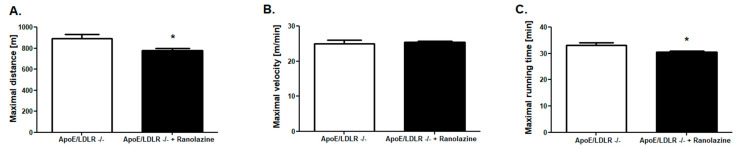
Increased exercise capacity in ApoE/LDLR -/- mice was blunted by Ranolazine (inhibitor of free fatty acids oxidation). Maximal distance (**A**), velocity (**B**), and running time (**C**) in incremental strenuous running test in ApoE/LDLR -/- and ApoE/LDLR -/- Ranolazine treated mice. Results presented as mean ± SEM, *n* = 10, * *p* < 0.05.

**Figure 9:**
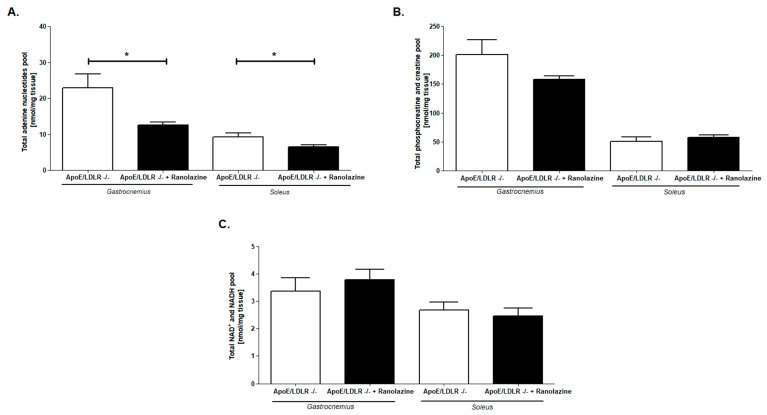
Enhanced total adenine nucleotides pool in skeletal muscles of ApoE/LDLR -/- mice was suppressed by Ranolazine (inhibitor of free fatty acids oxidation). Total adenine nucleotides (**A**), phosphocreatine and creatine (**B**), and NAD+ and NADH (**C**) pools in *gastrocnemius* and *soleus* of ApoE/LDLR -/-, and ApoE/LDLR -/- Ranolazine treated mice. Results presented as mean ± SEM, *n* = 6–8, * *p* < 0.05.

To test the role of free fatty acid (FFA) in exercise capacity improvement of ApoE/LDLR -/- mice, we inhibited the FFA oxidation with Ranolazine. We found that 7 days of Ranolazine treatment in 50 mg/kg body weight dose, resulted in the reduction of maximal distance and maximal running time in strenuous exercise protocol in ApoE/LDLR -/- mice (Figure 8A,C). No changes were noted in maximal velocity between those groups (Figure 8B). Ranolazine treatment leads also to the abolishment of the observed earlier enhanced total adenine nucleotides pool in *gastrocnemius* and *soleus* of ApoE/LDLR -/- mice (Figure 9A). On the other hand, no changes were noted in total phosphocreatine and creatine as well as NAD+ and NADH pools after Ranolazine treatment (Figure 9B,C).

### 2.5. Characterization of Fatty Acids Composition in ApoE/LDLR -/- Mice Serum

The last step of our research was the evaluation of fatty acids composition in ApoE/LDLR -/- mice serum in comparison to WT littermates (Table 1 and Table 2). Dyslipidemic mice serum were characterized by a reduced content of saturated fatty acids (SFA) such as lauric acid (C12:0), tridecanoic acid (C13:0), myristic acid (C14:0), pentadecanoic acid (C15:0), palmitic acid (C16:0), hexacosanoic acid (C26:0), as well as total SFA content. Interestingly one of the odd chains saturated FA, namely nonadecanoic acid (C19:0) exhibited reversed tendency. There were no changes in heptadecanoic acid (C17:0), eicosanoic acid (C20:0), heneicosanoic acid (C21:0), docosanoic acid (C22:0), tetracosanoic acid (C24:0), pentacosanoic acid (C25:0), and stearic acid (C18:0) serum content in comparison to control.

Analysis of monounsaturated FA (MUFA) shown reduced content of C22:1 (erucic acid), C24:1 (nervonic acid), C16:1 (palmitoleic acid) in ApoE/LDLR -/- mice serum, while other MUFAs contents such as C20:1 (11-Eicosenoic acid) and C18:1 ((11E)-11-octadecenoic acid) were elevated. C14:1 (myristoleic acid), C19:1 (nonadecenoic acid) as well as total MUFAs in ApoE/LDLR -/- mice serum content remain unchanged relative to WT. No changes were noted in polyunsaturated n-6 (PUFA n-6) and n-3 (PUFA n-3) serum content as well as total PUFA n-6 and n-3 pools.

We also calculated saturated to monounsaturated FA ratio (SFA/MUFA), saturated to polyunsaturated FA ratio (SFA/PUFA), and polyunsaturated n-6 to the polyunsaturated n-3 ratio (PUFAn-6/PUFA n-3) (Table 2). Double knock-out mice exhibited lower serum SFA/MUFA ratio than WT mice. There were no statistical differences between studied groups in SFA/PUFA and PUFA n-6/PUFA n-3 ratios.

## 3. Discussion

This study demonstrated the increased forelimb grip strength in the skeletal muscle in ApoE/LDLR -/- mice model. This functional enhancement was associated with increased troponin (I 1 and 2), and Mhc 2 levels, and the increased number of mitochondria in red skeletal muscles (indicated by enhanced citrate synthase activity). Furthermore, we noted elevation of adenine nucleotides pool, increased triglycerides, and decreased glycogen content in muscles of ApoE/LDLR -/-. Activated energy turnover in skeletal muscle seems to be related to an increase in serum-free fatty acids concentration. However, these functional and metabolic changes were specific only for ApoE/LDLR -/- and were not found in LDLR -/- mice. The important role of increased metabolism of free fatty acids was proven by the application of an inhibitor of fatty acid oxidation-Ranolazine in ApoE/LDLR -/- mice, which diminished improved outcome of exercise protocols and diminished total adenine nucleotides pool. We also underlined the reduced saturated fatty acids content and saturated to monounsaturated fatty acids ratio in ApoE/LDLR -/- mice serum.

Dyslipidemia is known to cause endothelial dysfunction, vascular alterations, restrictions in the oxygen supply, and organ dysfunction [10,12,13] and is an important element of metabolic syndrome (MS). MS relates to skeletal muscle dysfunction and metabolic derangements due to the accumulation of intramyocellular lipid intermediates (e.g., ceramides, diacylglycerols) and insulin resistance [14]. However, our previous work indicated that the insulin sensitivity was not altered in 6-month-old ApoE/LDLR -/- mice [9]. It seems that this stage of dyslipidemia is not yet causing these adverse effects in this experimental model. Moreover, we found that skeletal muscle substrate preference was switched from glucose to FFA, which is in excess in the serum of these mouse models [9]. Thus, they might also be able to produce the energy from elevated TG stores in skeletal muscle and in that way might suppress the negative effects of triglycerides accumulation in skeletal muscle.

High circulating free fatty acids levels besides direct contribution to oxidative metabolism may trigger regulatory cascades. There are findings indicating that FFAs may allosterically modulate AMP-activated protein kinase that it becomes a better substrate for the upstream kinase LKB1 [15,16]. Then, AMPK activation may lead to AMPK dependent PGC-1-α activation [17]. Skeletal muscle isolated from ApoE/LDLR -/- mice were characterized by both, AMPK as well as PGC-1α activation that may directly lead to stimulation of mitochondrial biogenesis [17]. Indeed, we found the increased activity of citrate synthase, a commonly used marker of mitochondrial abundance, in ApoE/LDLR -/- mice’s soleus muscle [18]. Enhanced mitochondria number may, therefore, compensate for the effect of reduced activity of mitochondrial complex II activity that in turn may relate to reduced levels of nuclear respiratory factor 1 (NRF-1) in double knock-out mice. NRF-1 is a transcriptional activator of nuclear genes that encode a range of mitochondrial proteins including cytochrome c and other respiratory chain subunits [19,20]. Ablation of NRF-1 in mice results in embryonic lethality due to loss of mitochondrial membrane potential and severely reduced mtDNA content, demonstrating NRF-1 as a potent regulator of mitochondrial function [21]. Moreover, NRF-1 activation via PGC-1α is necessary for proper mitochondrial biogenesis [22]. On the other hand, an isolated increase in NRF-1 is not sufficient to bring about a coordinated increase in the expression of all of the proteins necessary for the assembly of functional mitochondria [23]. It suggested that mitochondrial biogenesis stimulation might be a result of sustainable activation of all transcription factors related to this process. Moreover, the cause of observed NRF-1 levels might be its relation within the GLUT-4 expression. Skeletal muscle overexpression of nuclear respiratory factor 1 increases glucose transport capacity [23]. As mentioned earlier, ApoE/LDLR -/- mice’s skeletal muscle metabolism was shifted for fatty acids oxidation, thus metabolic pathways involved in glucose metabolism might be suppressed. Besides reduced NRF-1 levels, activation of mitochondrial biogenesis in ApoE/LDLR -/- mice might be sufficient not only to maintain a proper mitochondrial energy metabolism but also leads to its improvement, which was confirmed by enhanced total adenine nucleotides pool in investigated skeletal muscles.

AMPK and PGC-1α activation apart from energy metabolism improvement might lead to changes in myosin heavy chain gene expression and fiber type profile [24,25]. Mice carrying a mutation in the AMPKγ1 subunit leading to activation of AMPK show an increase in MyHC-2A and -2X in the triceps muscle with a corresponding increase in PGC-1α and citric synthase activity [24]. It is in the line with noted elevated levels of Mhc2 in gastrocnemius muscle isolated from ApoE/LDLR -/- mice. Furthermore, enhanced levels of troponin I type 1 (in slow skeletal muscle) as well as troponin I type 2 (in fast skeletal muscle) were noted, whose roles in skeletal muscle mechanics in skeletal muscle diseases are still underestimated [26]. Similar changes in troponins levels, total adenine nucleotide pools, and levels of transcription factors involved in energy metabolism in both types of investigated skeletal muscle: slow, red, *soleus* muscle, as well as fast, white, *gastrocnemius* muscle, explain the improvement in both, exercise capacity as well as forelimb grip strength in this dyslipidemic mice model. The other cause of observed forelimb grip strength improvement might be high glycogen content in skeletal muscle, nevertheless, this concept was overthrown after its examination in gastrocnemius of ApoE/LDLR -/- mice, glycogen stores were reduced.

As presented in our earlier research, both types of skeletal muscle exhibit metabolic preference shift to FA oxidation [9]. Interestingly, the Ranolazine treatment resulted in increased glucose use in cardiac metabolism and thus reduced FA β-oxidation [9,27]. It leads also to the abolishment of observed earlier improvement of exercise capacity in strenuous exercise protocol as well as reduction of total adenine nucleotides pool in gastrocnemius and soleus muscles. Thus, it unambiguously underpins the role of metabolic flexibility, serum-free fatty acid enhancement, and well as its increased oxidation in the improvement of function and metabolism of skeletal muscle under dyslipidemic conditions.

Changes in function and metabolism discussed above occurred in ApoE/LDLR -/- mice but not in LDLR -/- and control mice. ApoE/LDLR -/- mice fed normal diet develop robust hypercholesterolemia, with consequent endothelial dysfunction followed by atherosclerosis detected as early as at the age of 8 and 12 weeks, respectively [10,28,29]. LDLR -/- model shows a milder plasma cholesterol increase of 250 mg/dL when fed with a standard low-fat diet and the elevated lipoproteins are mainly in LDL class [30]. Atherosclerotic plaques are detectable not earlier than at the age of 6–12 months when mice are fed a normal diet [29,30,31] and atherosclerosis is usually accelerated by the Western diet. Here we confirmed that LDLR -/- mice were characterized by increased LDL and triglycerides but these changes were not such intensive in comparison to ApoE/LDLR -/- mice. Thus it might be suggested that mild dyslipidemia in the LDLR -/- mice model, does not induce adaptive changes in skeletal muscle function and metabolism as compared with robust dyslipidemia in ApoE/LDLR -/- mice. Moreover, ApoE/LDLR double knock-out mice strain differs significantly from WT mice and nonstatistical (but the unequivocal tendency was underlined) from LDLR -/- in FFA serum levels, which once again pronounced its possible role in skeletal muscle functionality and metabolic improvement.

It has to be mentioned that investigated mouse models are systemic knock-out, thus may not only have a direct impact on skeletal muscle function but also an indirect impact via signaling of the neuromuscular junction. Moreover, it is known that ApoE synthesis and secretion of cholesterol are regulated by the activity of ATP-Binding Cassette (ABC) transporters [32]. These ApoE containing particles are lipidated and become similar in size and density to peripheral high-density lipoproteins (HDL) and could be taken up by neurons via members of the LDLR family receptors, which recognize ApoE as a ligand [32]. LDLR-mediated uptake of ApoE-containing particles facilitates axonal and dendritic growth and is implicated in the recovery from peripheral nerve injury, during which excess cholesterol from membrane debris is cleared or redistributed to regenerating nerves [33]. In animal models of brain injury, ApoE and ABCA1 expression were increased, presumably to facilitate this transport [34]. Thus, the absence of these compounds may have a high impact on nervous system function.

The compositions of fatty acids in serum may have an impact on the development of metabolic disorders. The balance of saturated fatty acids (SFA) and unsaturated fatty acids (UFA) seems to be crucial due to their opposite effects on FA metabolism and inflammation. It is well known that SFAs induce metabolic disorders, whereas UFAs especially n-3 PUFAs may play a protective role [35,36]. The increase of SFAs in plasma is associated with low-grade inflammation in overweight adolescents [37]. We noted reduced total SFA as well as a trend to increased MUFA content in ApoE/LDLR -/- mice serum. Decreased SFA/MUFA ratio was also highlighted. The heightened MUFA content in serum is probably associated with the level of FFA and triacylglycerols. The majority of circulating FFA are released from triacylglycerols stored in adipose tissue, and MUFA is the most numerous fraction of FAs in adipose tissue [38]. Moreover, reduced SFA may be an effect of increased SFA desaturation in the liver and adipose tissue by stearoyl-CoA desaturase (SCD1) [38], but this needs to be verified in future studies. Reduced SFA and heightened MUFA might also suggest a preference for β-oxidation of SFA vs. MUFA which may result in more ATP molecules production and be the additional cause of the observed phenomenon. Additionally, Ragino et al. found that patients with atherosclerosis were characterized by increased MUFA levels and oleic acid which is in the line with our results [39]. They also found that higher levels of oleic acid may be associated with the relative risk of the presence of vulnerable atherosclerotic plaques in coronary arteries [39]. Interestingly, the content of one of SFA namely C19:0 was enhanced while others were reduced or exhibited no statistical differences. Moreover, there are some changes between dyslipidemic mice serum and atherosclerotic patients. Indeed, the content of palmitic (C16:0) or myristic acid (C14:0) in ApoE/LDLR -/- mice serum was diminished while atherosclerotic patients exhibited reverse tendency [39]. In addition, interesting patterns of MUFAs content were noted. 11-Eicosenoic acid (C18:1) and C20:1 acid were increased, while the content of longer MUFA—C22:1, C24:1 was reduced in ApoE/LDLR -/- mice serum. The possible explanation of this observation may be the reduced activity of elongase 1 (ELOVL1) [40] that is responsible for elongation of very long-chain MUFAs, and this is another indication that FA metabolism in liver and adipose tissue requires further study in this experimental model. The examination of the role of FA content in improved cardiac and skeletal muscle function in this experimental model requires further studies.

**Figure 10:**
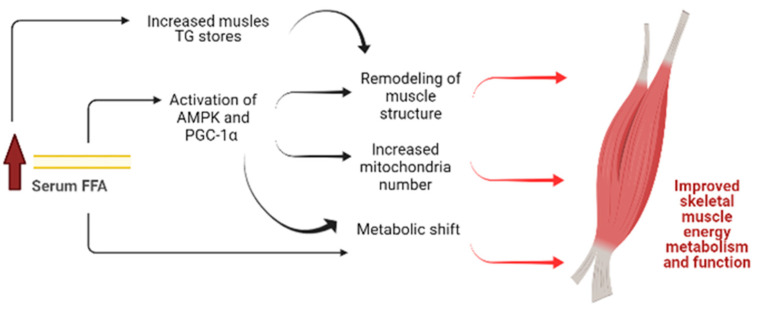
A model depicting the mechanism by which increased serum free fatty acids (FFA) concentration may lead to improvement of skeletal muscle metabolism and function in ApoE/LDLR -/- mice. Increased FFA levels led to 1. Elevation of skeletal muscle triglycerides stores and 2. Activation of AMPK as well as PGC- 1α signaling pathways. Those factors may induce remodeling of muscle structure. Enhanced AMPK phosphorylation and PGC- 1α levels activated the mitochondrial biogenesis that resulted in increased mitochondria number. Heightened FFA levels accompanied with AMPK activation led to metabolic shift resulting in increased skeletal muscle FFA oxidation. These alterations may contribute to improved skeletal muscle energy metabolism and thus enhanced grip strength and physical performance of ApoE/LDLR -/- mice. The hypothesis was supported by the demonstration that inhibition of FFA oxidation via Ranolazine resulted in depletion of skeletal muscle total nucleotides pool as well as the abolishment of improved mice exercise capacity. Created with Bioreder.com at 2 November 2021.

In summary, the ApoE/LDLR -/- mice model at the age of 6 months displays a number of unexpected alterations in skeletal muscle function and metabolism that may contribute to enhanced grip strength and physical performance reported here and previously (Figure 10) [8,9]. Remarkable adaptation to dyslipidemic conditions surprisingly turns into an asset thanks to metabolic switching to intensify fatty acids metabolism. This is consistent with our earlier observations of such metabolic adaptations with no derangements in the functionality of cardiac muscle [9]. However, it has to be stressed that this phenomenon might be age-related and could turn from adaptive to maladaptive in older ApoE/LDLR-/- mice. Caution is necessary to avoid the deleterious effects of increased lipid availability; however, such a strategy of metabolic adaptation in skeletal muscle as reported here in ApoE/LDLR-/- mice when reproduced by pharmacological treatment may help to improve skeletal muscle function in specific pathological scenarios.

## 4. Limitations

It is crucial to note that the presented work is focused mainly on the evaluation of skeletal muscle function and metabolism and does not include the skeletal muscle structure assessment using histological analysis. Morphological evaluation of changes in skeletal muscle fibers architecture, mitochondria quality, or presence of cellular inclusions might be an important aspect of dyslipidemic skeletal muscle analysis. However, while there is no study ApoE/LDLR -/- such analysis in LDLR -/-mice failed to demonstrate any morphological changes or accumulation of lipid [41].

Our research tested only two dyslipidemic mouse models which may not give a comprehensive insight into skeletal muscle metabolism and function in diverse mechanisms of dyslipidemia. Analysis of skeletal muscle structure, metabolism, and function of another known dyslipidemic mice model, single knock-out- ApoE -/- demonstrated lower exercise capacity, a decline in maximum oxygen uptake, and an anaerobic threshold [42]. Furthermore, delayed maturation of skeletal muscle decreased systemic expression of cytokines, and growth factors were found [43]. Analysis of skeletal muscle structure in ApoE -/- mice revealed conflicting results. One study found accumulation of phospholipids in the form of sarcoplasmic reticulum tubules in the type 2 fibers and enlarged mitochondria with tightly packed cristae in the type 1 fibers [44], while the other indicated the absence of any muscle pathology in ApoE -/- mice treated with a normal Western diet [45].

Despite these limitations, our study for the first time highlighted that contrary to the common view, dyslipidemia with increased free fatty acid concentration may lead to accelerated fatty acids oxidation and improved skeletal muscle function, at least at a specific stage of dyslipidemic pathology. This may have implications for understanding and developing manipulations with lipid patterns as a treatment. 

## 5. Materials and Methods

### 5.1. Animals

All experiments were conducted following the Guide for the Care and Use of the Laboratory Animals published by the European Parliament, Directive 2010/63/EU, and were approved by the local bioethical committee for the Medical University of Gdansk. Animals were maintained on a 12:12 h light–dark cycle at 25 °C, 30–40% humidity, and were provided with free access to water and a standard chow diet. Six-month-old ApoE/LDLR -/- (*n* = 37), LDLR -/- (*n* = 18), and C57BL/6J (*n* = 35) as WT mice were used in the study. Evaluation of skeletal muscle grip strength, skeletal muscle protein levels as well as muscle mitochondrial chain complexes activities in dyslipidemic and their WT littermates (ApoE/LDLR -/- vs. WT and LDLR -/- vs. WT) were conducted as separate, independent experiments. While in the investigation of total adenine nucleotides pool, TG and glycogen content in skeletal muscles as well as serum glucose, FFA, and lipid profile were examined at the same time in three experimental groups (WT, LDLR -/-, and ApoE/LDLR -/-). It resulted from the complexity of the mentioned analyzes and the availability of animals in selected age.

#### 5.1.1. Forelimb Grip Strength Measurement

Forelimb grip strength was measured by a grip strength meter (GSM Grip strength meter, Ugo Basile, Gemonio VA, Italy). The animal was held so that only the forelimb paws grasped the specially designed mouse flat mesh assembly and the mouse was pulled back until their grip was broken. The force transducer retained the peak force reached when the animal’s grip was broken, and this was recorded from a digital display. Five successful forelimb strength measurements within 2 min were recorded. The maximum values were used for analysis. The mice were trained on the grip strength meter before the trial. Forelimb and maximal muscle strength were obtained as values of GF (gram-force) and normalized to bodyweights as “g/g mouse body weight.”

#### 5.1.2. Ranolazine Treatment and Evaluation of Exercise Capacity

ApoE/LDLR -/- was treated daily with 50 mg/kg of Ranolazine or 0.9% NaCl administered intraperitoneally for one week as described earlier [9]. After the treatment mice were assigned to a single bout of strenuous exercise on a treadmill according to a protocol described in our previous works [9,11].

#### 5.1.3. Skeletal Muscle Isolation and Serum Collection

Mice were anesthetized with ketamine/xylazine mixture (50 mg/kg + 5 mg/kg), underwent artificial ventilation, skeletal muscle (*gastrocnemius* and *soleus*) isolation, and freeze-camping. Blood was collected from *Inferior vena cava* (*IVC*) after animal anesthesia. For serum collection, blood was centrifuged at 2000 RPM for 4 min.

### 5.2. Evaluation of Skeletal Muscle Protein Levels

Analysis of skeletal muscle levels of troponins (I Type 1, I Type 2), myosin heavy chain 2 (Mhc2), nuclear respiratory factor 1 (NRF-1), peroxisome proliferator-activated receptor-gamma coactivator 1 alpha (PGC-1 alpha), and phosphorylated form of adenosine monophosphate-activated protein kinase (AMPK), were performed using ELISA Kits (Abcam, Cambridge, UK; Qayee Bio-Technology C, Shanghai, China; Wuhan EIAab Science Co, Wuhan, China) according to the manufacturer’s instructions.

### 5.3. Investigation of Skeletal Muscle Mitochondrial Chain Complexes Activities

Mitochondria were isolated from *soleus* muscle based on the previously described procedure [46]. After skeletal muscle isolation, soleus muscle was put into iced-cold isolation Buffer (70 mM sucrose, 210 mM mannitol, 5 mM HEPES, 1 mM EGTA with 0.5% (*w*/*v*) fatty acid-free BSA (pH 7.2) and homogenized. Then, the homogenate was centrifuged for 10 min at 500× *g*, 4 °C for 10 min. After first centrifugation, fat was carefully discarded, and the remaining supernatant was transferred to a separate tube and centrifuged once again at 10,000× g for 10 min at 4 °C. Pellet was resuspended in 1 mL of Isolation Buffer without BSA to determine the mitochondrial protein concentration using Bradford Assay reagent (BioRad). The next step of the analysis was performed by Seahorse Metabolic Flux Analyzer (Agilent Technologies). For electron flow experiments, isolated mitochondria were diluted in cold MAS buffer enriched with 10 mM pyruvate, 2 mM malate, and 4 µM FCCP. 25 µL mitochondrial suspension was placed into Seahorse plate wells and centrifuges at 2000× *g* for 15 min at 4 °C. The concentration of mitochondrial protein was 6 µg per well. After centrifugation, 180 µL of prewarmed MAS buffer supplemented with pyruvate, malate, and FCCP was added to each well, and the plate was then placed into a non-CO2 incubator for 8 min. In the meantime, the Seahorse cartridge was filled with the following reagents: 2 µM Rotenone, 2 mM succinate, 4 µM Antimycin, and a mix of 10 mM ascorbate and 100 µM TMPD. 

### 5.4. Measurement of Total Phosphocreatine and Creatine, Nicotinamide Dinucleotides, and Adenine Nucleotides Pool

Skeletal muscles were freeze-clamped, prepared, and analyzed with the high-pressure liquid chromatography (HPLC) method as previously described [47].

### 5.5. Evaluation of Skeletal Muscle Triglycerides, Glycogen Stores, and Citric Synthase Activity

Muscles triglycerides and glycogen stores were measured using colorimetric assays according to manufacturer instructions (BioVision, CA, USA and Abcam, Cambridge, UK). Briefly, for triglycerides quantification *soleus* muscles were homogenized in 5% NP-40 then samples were repeatedly heated and cooled down to solubilize all triglycerides and directly analyzed within assay procedure. For skeletal muscle glycogen storage measurement, *gastrocnemius* muscles were homogenized in distilled water in 10 mg tissue per 200 µL of water ratio and centrifuged at 1800 RPM for 10 min. The obtained supernatant was used in the analysis. Triglycerides level in skeletal muscle was presented as nmol/mg tissue while glycogen stores as µg/mg tissue.

Citric synthase activity in *soleus* was measured within the assay kit (Sigma-Aldrich, MO, USA). The activity of the enzyme is measured by following the color of 5-thio-2-nitrobenzoic acid (TNB), which is generated from 5,5′-Dithiobis-(2-nitrobenzoic acid) (DTNB) present in the reaction of citrate synthesis, and caused by the deacetylation of Acetyl-CoA. The overall reaction product, TNB, absorbs at 412 nm. Citric synthase activity in the *soleus* muscle was presented as µmol/mg tissue/min.

### 5.6. Investigation of Serum-Free Fatty Acids, Glucose, and Lipid Profiles

The FFA concentration in serum was measured using a commercial colorimetric assay kit (Wako NEFA C test kit; Wako Chemicals, Neuss, Germany). While the lipid profile and glucose concentrations in serum were determined using an automatic biochemistry analyzer (Erba XL-180, Erba Mannheim GmbH, Mannheim, Germany). Serum was collected after 24-h starvation.

### 5.7. Fatty Acids Serum Composition 

The fatty acids serum composition was measured by gas chromatography-mass spectrometry as described earlier [48]. Total lipids were extracted from mice serum with a mixture of chloroform and methanol (2:1, *v*/*v*) according to Folch et al. [49]. Next, a total lipid extract from each sample was subjected to 3 h of hydrolysis with 0.5 M KOH at 90 °C. After incubation, the mixtures were acidified with 6 M HCl. One milliliter of water was added, and unesterified FAs were extracted thrice with 1 mL of n-hexane and the organic phase was evaporated under a nitrogen stream. Extracts were then derivatized into fatty acid methyl esters (FAME) with 10% boron trifluoride in methanol solution at 55 °C for 1.5 h. Then, 1 mL of water was added, and FAME were extracted with 3 × 1 mL n-hexane, dried under nitrogen stream and stored at −20 °C until analysis.

FAME was analyzed on GC-EI-MS QP-2010SE (Shimadzu, Kyoto, Japan) with chromatographic separation on Zebron ZB-5MSi capillary column, 30 m × 0.25 mm i.d. × 0.25 µm film thickness, (Phenomenex, Torrance, CA, USA). Separation parameters were set as follows: column oven temperature 60–300 °C (4 °C/min), total analysis run time was 60 min; helium was used as a carrier gas (column head pressure at 100 kPa). MS analysis was conducted in full scan mode, with the mass scan range set at m/z 45–700. Electron impact source was operating at 70 eV. FAs were identified using reference standards mixture (37 FAME Mix, Sigma Aldrich, St. Louis, MO, USA) and reference library NIST 11. The internal standard was 19-methylarachidic acid.

### 5.8. Statistical Analysis

Statistical significance was evaluated using Student’s t-test for comparatives of two groups while one-way ANOVA with Bonferroni correction was used while three groups were tested. A value of *p* < 0.05 was used to denote statistical significance, and results are expressed as mean ± SEM. All statistics were carried out using GraphPad Prism 5.00 (GraphPad Software, San Diego, CA, USA).

## Figures and Tables

**Table 1:** Reduced saturated fatty acids and increased monounsaturated fatty acids content in ApoE/LDLR -/- mice serum. Results presented as mean ± SEM, *n* = 6, * *p* < 0.05, ** *p* < 0.01, *** *p* < 0.001.

Parameter [%]	WT	ApoE/LDLR -/-	*p*
C10:0	0.10 ± 0.003	traces	-
C12:0	0.30 ± 0.04	0.04 ± 0.004	***
C13:0	0.03 ± 0.003	0.01 ± 0.002	***
C14:0	1.03 ± 0.10	0.22 ± 0.04	***
C15:0	0.51 ± 0.05	0.24 ± 0.01	**
C16:0	24.12 ± 1.53	18.05 ± 0.62	*
C17:0	0.47 ± 0.03	0.56 ± 0.05	n/s (0.13)
C18:0	9.78 ± 1.08	8.37 ± 0.24	n/s (0.3)
C19:0	0.16 ± 0.008	0.22 ± 0.011	**
C20:0	0.27 ± 0.04	0.29 ± 0.03	n/s (0.7)
C21:0	0.05 ± 0.007	0.04 ± 0.003	n/s (0.1)
C22:0	0.30 ± 0.04	0.21 ± 0.02	n/s (0.07)
C24:0	0.30 ± 0.03	0.20 ± 0.01	n/s (0.051)
C25:0	0.06 ± 0.009	0.06 ± 0.009	n/s (0.9)
C26:0	0.04 ± 0.008	0.01 ± 0.001	*
Total SFA	37.51 ± 2.69	28.57 ± 0.52	*
C14:1	0.02 ± 0.005	0.02 ± 0.003	n/s (0.6)
C16:1	4.26 ± 0.33	3.10 ± 0.10	**
C18:1	18.93 ± 1.45	24.78 ± 0.79	**
C19:1	0.01 ± 0.003	0.01 ± 0.002	n/s (0.4)
C20:1	0.34 ± 0.03	1.04 ± 0.10	***
C22:1	0.51 ± 0.08	0.10 ± 0.02	**
C24:1	0.60 ± 0.09	0.34 ± 0.03	*
Total MUFA	24.67 ± 1.76	29.38 ± 0.74	n/s (0.07)
C16:2n-6	0.04 ± 0.008	0.05 ± 0.01	n/s (0.6)
C18:2n-6	25.72 ± 1.57	29.90 ± 0.95	n/s (0.07)
C20:2n-6	0.09 ± 0.01	0.1 ± 0.008	n/s (0.6)
C20:3n-6	0.56 ± 0.075	0.53 ± 0.053	n/s (0.7)
C20:4n-6	6.12 ± 1.39	5.54 ± 0.42	n/s (0.7)
C22:4n-6	0.04 ± 0.005	0.05 ± 0.004	n/s (0.3)
Total PUFA n-6	32.57 ± 2.91	36.16 ± 1.01	n/s (0.3)
C18:3n-3	0.23 ± 0.71	0.21 ± 0.04	n/s (0.8)
C20:4n-3	0.05 ± 0.009	0.03 ± 0.004	n/s (0.2)
C20:5n-3	1.10 ± 0.33	1.85 ± 0.24	n/s (0.1)
C22:5n-3	0.34 ± 0.07	0.48 ± 0.06	n/s (0.2)
C22:6n-3	3.54 ± 0.78	3.27 ± 0.23	n/s (0.2)
Total PUFA n-3	5.26 ± 1.19	5.85 ± 0.44	n/s (0.6)

**Table 2:** Enhanced SFA/MUFA ratio in ApoE/LDLR -/- mice serum. Results presented as mean ± SEM, *n* = 6, * *p* < 0.05, *** *p* < 0.001.

Parameter	WT	ApoE/LDLR -/-	*p*
SFA/MUFA ratio	1.52 ± 0.09	0.97 ± 0.02	***
SFA/PUFA ratio	0.99 ± 0.20	0.68 ± 0.02	n/s (0.1)
PUFA n-6/PUFA n-3 ratio	6.19 ± 2.50	6.18 ± 0.63	n/s (0.9)

## Data Availability

The authors declare that the data supporting the findings of the study are available within the article.
